# Effects of the COVID-19 Pandemic on Anxiety and Depression among Medical Interns

**DOI:** 10.5811/westjem.38455

**Published:** 2025-07-13

**Authors:** Tugay Usta, Serap Biberoğlu, Afşin İpekci, İbrahim İkizceli, Fatih Çakmak, Yonca S. Akdeniz, Gülçin Baktıroğlu, Seda Özkan

**Affiliations:** * Kanuni Sultan Suleyman Training and Research Hospital, Department of Emergency Medicine, Istanbul, Türkiye; † İstanbul University Cerrahpaşa, Cerrahpaşa Faculty of Medicine, Department of Emergency Medicine, Istanbul, Türkiye

## Abstract

**Introduction:**

The demanding nature of emergency medicine (EM), requiring immediate responses to emergencies, and presents significant challenges, particularly for new trainess specialty. Our goal was to evaluate levels of anxiety and depression among EM intern doctors, with focus on the impact of the COVID-19 pandemic.

**Methods:**

We conducted this study at Istanbul University-Cerrahpasa, Department of Emergency Medicine, from December 29, 2019–May 2, 2021. In Türkiye, the six year medical education program has the first three years preclinical, the fourth and fifth years comprised of clerkships, and the sixth year is internship training. In this final year, these intern doctors rotate through various departments, including an 8-week EM internship. A total of 203 medical interns participated in the study, 50.2% male. We assessed participants using the State-Trait Anxiety Inventory (STAI 1–2) and the Beck Depression Inventory, both prior to starting their EM internship and upon completion. Intern doctors were divided into two groups: 51 who completed their internship before the COVID-19 pandemic (December 29, 2019–March 11, 2020) and 152 during the pandemic (March 11, 2020–May 2, 2021). We compared pre- and post-internship scores within each group and between the two cohorts.

**Results:**

Anxiety scores (STAI-1) increased significantly in both groups during the internship. In the pre-COVID-19 group, median STAI-1 scores rose from 47 (IQR: 38–53) to 51 (IQR: 45–56) (p<0.001), and in the COVID-19 group, from 41 (IQR: 35–48) to 47 (IQR: 42–52) (p<0.001). However, depression scores (BDI) showed a significant increase only in the pre-COVID-19 group: from 9 (IQR: 2–14) to 26 (IQR: 15–32) (p<0.001). In contrast, the COVID-19 group’s depression scores remained relatively stable, increasing only from 7 (IQR: 2–13) to 8 (IQR: 3–16) (p=0.345).There were no significant differences between the groups in trait anxiety (STAI-2) scores (p=0.221) or pre-internship BDI scores (p=0.408). However, post-internship BDI scores were significantly lower in the COVID-19 group compared to the pre-COVID-19 group (median: 8 vs. 26; p<0.001).

**Conclusion:**

The EM internship was associated with an increase in anxiety levels among intern doctors. Depression scores did not show a significant increase in the COVID-19 group, whereas depression scores significantly increased in the pre-COVID-19 group by the end of the internship. These findings suggest that, while anxiety increased across both groups, depression levels were more stable in the COVID-19 group, with lower post-internship scores compared to those in the pre-COVID-19 group.

## INTRODUCTION

Emergency medicine (EM) is a rapidly evolving specialty that addresses and manages acute medical conditions across various disciplines. The demanding nature of this field, requiring immediate responses to emergencies of varying urgency, presents significant challenges, particularly for individuals new to the specialty. Unlike postgraduate trainees in EM in other countries, intern doctors in Turkey undergo practical training across multiple specialties during their sixth and final year of medical education. These challenges are particularly prominent for interns as they rotate through different departments, including EM. Numerous studies have highlighted the physical and psychological toll on them, emphasizing their experiences of anxiety, depression, and overall stress while working in the high-pressure environment of EM rotations.^1^–^7^

Anxiety disorder is characterized by an emotional state of unprovoked anxiety, accompanied by distress and restlessness. This condition can manifest through worries spanning various aspects of life, including health, work, interpersonal relationships, and daily activities, often leading to physical symptoms according to the *Diagnostic and Statistical Manual of Mental Disorders*, 5^th^ *Ed* (DSM-5). 8 Such anxiety can disrupt daily functioning, impairing sleep, appetite, and overall quality of life. 9 Additionally, anxiety is closely associated with depression and an increased risk of suicide. 2,10 The incidence of mental health issues and substance use disorders has risen as a result of anxiety.^5^, ^11^

Depression is a mood disorder marked by persistent feelings of sadness, hopelessness, and a lack of interest in activities previously enjoyed. Depression is characterized by a range of symptoms, including feelings of worthlessness, fatigue, changes in sleep and appetite, and difficulty concentrating. These symptoms can significantly impair an individual’s daily functioning and well-being.^8^ Depression not only affects the individual but can also compromise the quality of care provided to patients.^12^ Physicians suffering from depression, for instance, have been shown to be 6.2 times more likely to make medication errors than their non-depressed counterparts.^13^

Studies focusing on healthcare professionals have consistently highlighted the substantial stress faced by those in the field, with emergency clinicians experiencing particularly high levels of stress compared to other occupational groups.^7^ It has also been reported that physicians working in emergency departments have a higher propensity for alcohol and substance use disorders than the general population, often as a means of coping with escalating depression levels.^14^,^15^

A search of PubMed revealed a notable gap in research addressing the prevalence of anxiety and depression among interns. Despite the publication of 60 studies related to anxiety among EM interns and 83 studies on depression, there remains limited research focusing specifically on the well-being of interns during their EM rotations. We investigated the prevalence of anxiety and depression among intern doctors at Istanbul University-Cerrahpaşa Faculty of Medicine, with a specific focus on comparing the levels of these conditions both before and after completing their eight-week EM internship.

We further evaluated the impact of internship year on the participants’ anxiety and depression levels. Given the emergence of the COVID-19 pandemic during the study, we also wanted to examine how the pandemic may have influenced these outcomes. By comparing data from both the pre-pandemic and pandemic periods, this research provides insights into the challenges faced by intern doctors and contributes to the existing literature. Our findings help clarify whether the pandemic exacerbated or altered the levels of anxiety and depression among intern doctors.

**Population Health Research Capsule**

What do we already know about this issue?

*Anxiety and depression are serious but largely unrecognized issues among interns in emergency medicine*.

What was the research question?

*Does the internship year elevate anxiety and depression levels in intern doctors?*

What was the major finding of the study?

*We found significant increases in the Beck Depression Inventory (P < .001, CI 1.032–1.079) and the State-Trait Anxiety Inventory I (P < .001, CI 1.037–1.083)*.

How does this improve population health?

*This study highlights the psychological impact of EM internship and the need for mental health support to improve medical interns’ well-being*.

## MATHERIAL AND METHODS

### Ethics and Consent

This study was conducted in accordance with the ethical principles outlined in the Declaration of Helsinki. It was approved by the Clinical Research Ethics Committee of Istanbul University-Cerrahpasa Faculty of Medicine (approval number: 83045809-604.01.02, date: 24/03/2020). Written informed consent was obtained from all participants before we began to collect data.

### Study Design and Setting

This prospective cohort study was conducted at Istanbul University-Cerrahpasa Faculty of Medicine, Department of Emergency Medicine. The primary objective was to evaluate the effects of the EM internship on anxiety and depression levels in final-year medical students during the COVID-19 pandemic. Outcomes were measured using validated psychological tools to assess state and trait anxiety, as well as depressive symptoms. State anxiety refers to a person’s temporary emotional response to stress, while trait anxiety reflects a more permanent predisposition to be anxious.

### Outcomes

- Primary outcomes: Changes in anxiety (measured by the State-Trait Anxiety Inventory 1 and 2 [STAI]) and depression (measured by the Beck Depression Inventory [BDI]) scores from pre-internship to post-internship, comparing the pre-COVID-19 and COVID-19 groups.
- Secondary outcomes: Changes in trait anxiety (measured by STAI-2) scores pre-internship, as well as post-internship changes in depression and anxiety scores (STAI-1, BDI) by sex (female vs male).

### Participants and Recruitment

Participants were final-year medical student/intern doctors completing their mandatory EM internship between December 29, 2019–May 2, 2021. The EM internship is a required component of the medical curriculum, lasting eight weeks, during which interns typically work 16 day shifts and 16 night shifts. During their internship, intern doctors were responsible for assessing patients, history-taking, assisting in emergency procedures, and participating in patient care under the supervision of attending physicians. The duties of intern doctors became more varied during the pandemic, with increased telemedicine consultations and less direct patient contact.

The first COVID-19 case in Türkiye was reported on March 11, 2020. Based on this date, intern doctors were divided into two groups: the pre-COVID-19 period (December 29, 2019–March 11, 2020) and the COVID-19 period (March 11, 2020–May 2, 2021). Cases of COVID-19 peaked in late 2020/early 2021, and reached the highest spike between December 2021–February 2022, followed by a sharp decline.^16^ To minimize the risk of COVID-19 exposure, the shift schedules of interns were temporarily reorganized into eight extended 24-hour shifts during the pandemic period.

Recruitment involved inviting all intern doctors assigned to the emergency department (ED) during the study period to participate. Recruitment emails were sent out twice: one initial invitation and a follow-up reminder a week later. Participation was voluntary, and no incentives were provided. Of 600 eligible intern doctors, 450 consented to participate in the study (75% participation rate). After excluding 97 individuals due to pre-existing psychiatric conditions or inability to perform night shifts and an additional 150 due to incomplete data, the final analysis included 203 intern doctors (33.8% of the initial eligible population).

Exclusion criteria for the study were as follows: interns who declined participation; were unable to maintain their scheduled shifts (either day or night) during the EM internship due to chronic illness; individuals with substance use disorder, and those with known history of psychiatric disorders. Although anxiety and depression are included as psychiatric disorders in the DSM-5,^8^ we made this exclusion to enhance the validity of the study and specifically examine the effects of medical education-related stress. Substance use and psychiatric disorders are often linked to stress in medical students and may introduce confounding variables that affect anxiety and depression levels.^17^,^18^ Including participants with these conditions could have complicated the analysis by introducing factors unrelated to educational stress, making it difficult to isolate the specific impact of medical education on anxiety and depression levels. Therefore, to ensure a clear focus on the effect of educational stress on anxiety, we excluded participants with substance use or psychiatric disorders.

### Psychological Assessments

We used the BDI and the STAI-1 and STAI-2 to measure psychological outcomes:

1. The BDI is a 21-item questionnaire measuring depressive symptom severity, taking approximately 10 minutes to complete. Scores classify symptoms as minimal, mild, moderate, or severe. We used a validated Turkish version of the BDI^19^ administered before and after the internship to evaluate immediate changes in depression levels.
2. The STAI-1 and STAI-2 consists of two subscales: STAI-1 assesses state anxiety (temporary emotional responses to stress), while STAI-2 evaluates trait anxiety (a person’s general tendency to experience anxiety). Each subscale contains 20 items, rated on a 4-point scale, with adjustments for positively and negatively worded items. Final scores range from 20–80, with higher scores indicating greater anxiety levels. The average anxiety score typically falls between 36–41. The Turkish version has been validated for use in research and clinical settings.^20^

These tools were chosen over alternative scales such as the Patient Health Questionnaire-9 (PHG-9) and Generalized Anxiety Disorder-7 scale (GAD-7) based on their validity, reliability, and previous use in similar research contexts.^19^–^24^ The BDI and STAI-1 were administered to assess fluctuations in depressive symptoms and state anxiety throughout the internship, while STAI-2 was used to measure trait anxiety, providing a more stable evaluation of baseline anxiety levels.

### Timing of Assessments

Participants completed the STAI-1, STAI-2, and BDI assessments two days before starting their first shift and within two days after completing their final shift. This timing ensured that the measurements reflected changes specifically related to the internship experience.

### Confidentiality

All data were anonymized, and only the research team had access to the assessments. Faculty members in the ED were not informed of participants’ involvement in the study to prevent potential bias or coercion.

### Sample Size Calculation

A power analysis using the G*Power 3.1 software (Heinrich Heine University Düsseldorf, Germany) determined that a minimum of 179 participants was required to detect a medium effect size (d = 0.25) with 90% power and a 5% significance level. The final sample size of 203 participants exceeded this threshold, ensuring adequate statistical power.

### Statistical Analysis

We conducted all statistical analyses were conducted using SPSS Statistics 22.0 (SPSS Statistics, IBM Corp, Armonk, NY). Continuous variables were summarized as medians and interquartile ranges (IQR) for non-normally distributed data and means with standard deviations for normally distributed data. Categorical variables were presented as frequencies and percentages.

1. Normality testing: We evaluated data distribution using the Kolmogorov-Smirnov and Shapiro-Wilk tests, along with skewness and kurtosis assessments.
2. Comparisons: For normally distributed variables, we used the Student t-test. For non-normally distributed variables, the Mann-Whitney U test or Kruskal-Wallis test was applied as appropriate.
3. Categorical variables: We assessed differences using the Pearson chi-square test or Fisher exact test, where applicable.
4. Significance level: A *P*-value < .05 was considered statistically significant.

## RESULTS

During the study period, a total of 600 intern doctors were eligible to participate. Of these, 450 provided informed consent, resulting in a 75% participation rate. After excluding interns with psychiatric disorders and other ineligibility criterias, the final analysis included 203 intern doctors, representing 33.8% of the initial eligible population (Table 1).

Of the 203 intern doctors who participated in our study, 49.8% were female and 50.2% were male. The median age of males and females was 23 (23–24). Interns scored 44 (38–51) on the STAI-2 scale and showed moderate anxiety; significantly higher anxiety was observed in females than in males (*P*<.001). It was determined that there was no difference between the sexes in STAI-1 both before (*P*=.13) and after (*P*=0.23) the internship Before the EM internship, moderate anxiety was observed with a score of 43 (36–49) on the STAI-1 scale. At the end of the internship, a high degree of anxiety was observed with a score of 48. Male (*P*<.001), female (*P*<.001) and total (*P*<.001) had a statistically significant increase in anxiety levels.[Fig f1-wjem-26-795]

Before their EM internship, interns scored 7 (2–14) on the BDI scale and showed minimal depression; significantly higher depression was observed in females compared to males (*P*<.001). At the end of the internship, they scored 11 (4–23), demonstrating mild depression, with levels of depression in females still significantly higher than in males (*P*=.035). Depression levels increased statistically significantly in males (*P*<.001), females (*P*=.03), and total (*P*<.001). Table 2 shows the depression and anxiety levels of intern doctorss before and after their EM internship according to the STAI-1 and BDI, and their distribution according to their percentages is given. Statistically significantly increased depression was observed in males (*P*<.001), females (*P*=.01), total (*P*<.001). Statistically significantly increased anxiety was observed in males (*P*<.001), females (*P*<.001), total (*P*<.001). It was observed that COVID-19 did not lead to a significant change in STAI-2 and BDI scores before the EM internship (*P*=.08, *P*=.22). However, after the EM internship, the median BDI score was 26 (IQR 15–32) before the COVID-19 period, indicating moderate depression, whereas after COVID-19, the median score decreased to 8, reflecting minimal depression. This reduction in depression levels was statistically significant (*P*<.001). Before the EM internship, intern doctors had high anxiety levels, with a median STAI-1 score of 47 (IQR 38–53). After the internship, anxiety remained high, with a median score of 51 (IQR 45–56). During the COVID-19 period, anxiety levels were lower both before and after the internship, with median scores of 41 (IQR 35–48) and 47 (IQR 42–52), respectively. These reductions were statistically significant (*P* = .02). Table 4 summarizes multivariate regression analysis we conducted while controlling for sex and COVID-19 process factors, which we identified as potential confounding variables in our study, using logistic regression. The results indicated that BDI and STAI-1 scores were associated with increases during the EM internship (*P*<.001). These findings suggest a relationship between the internship and changes in the scores, although causality could not be definitively established.

## DISCUSSION

In this study our goal was to compare the anxiety and depression levels of interns at the beginning and end of their EM internships and to determine the impact of the internship on these parameters. Given that the study period coincided with the COVID-19 pandemic, we anticipated that the pandemic could have had a confounding effect, and we sought to identify and control for this influence. Our findings indicate a significant increase in both anxiety and depression scores throughout the internship, with differences observed between male and female interns. While both sexes experienced a notable rise in anxiety and depression levels, the increase in anxiety was more pronounced among male interns, whereas depression levels showed a comparable rise across sexes. Interestingly, COVID-19 pandemic reduced anxiety and depression levels among intern doctors by the end of their EM internships.

Ina study conducted by Aslan et al in which 358 students from 14 universities participated, using the GAD-7, the Satisfaction with Life Scale, the Perceived Stress Scale, and the Physical Activity Scale. In 52% of the students, they found generalized anxiety disorder and major depression in 63% of them and that females and physically inactive students were more anxious and depressive than their counterparts.^25^

Although different assessment tools were used compared to our study, similar sex-related findings were observed. The reasons behind these findings remain unclear; however, a study conducted in Korea examining adolescent populations also found that females exhibited higher levels of depression than males. In their study, the authors suggested that hormonal factors, emotional burden, parenting styles, and communication problems could contribute to this sex disparity in depression.^26^ These factors may also help explain the differences observed in our study in BDI and STAI-2.[Table t3-wjem-26-795]

Importantly, no significant difference was observed between male and female intern doctors in STAI-1 scores, both before and after the EM internship. This finding may be attributed to the nature of situational anxiety, which is often triggered by acute stressors that affect individuals regardless of their sex. The high-stress environment of EM, characterized by unpredictable cases, critical decision-making, and fast-paced conditions, may have created a uniformly stressful experience for all interns, thus overshadowing potential sex-related differences.^27^

In such high-pressure situations, the immediate, situational stress may not be as differentiated by sex, as both male and female interns may exhibit similar coping mechanisms and stress responses to acute stressors in the short term.^12^ However, this contrasts with trait anxiety (STAI-2), which reflects more enduring, personality-related tendencies. Sex differences in trait anxiety are often more pronounced, with females generally exhibiting higher baseline anxiety levels than males.^28^ This suggests that while females may have higher predispositions toward anxiety, the intense and immediate stressors of the EM environment could exert a more equalizing effect on situational anxiety in males and females.^29^

Depression, Anxiety, and Stress Scales were applied to the participants at the beginning and end of the internship to determine the effect of EM internship on the levels of anxiety and depression in EM service internship.^3^ However, another study conducted by Erdur et al on emergency physicians found that as the time spent in the ED increased, depression and anxiety increased, and our findings aligns with their results.^30^ The chaotic nature of the ED, exacerbated by witnessing events such as patients who died, working in the ED for the first time in a role of responsibility, increased workload, worries about the future, and night shifts may have made interns more anxious.^30^–^34^

Another important reason for increased anxiety is the incidence of violence in healthcare. It has been reported that violence against healthcare workers globally, as well as in Türkiye, is a serious occupational hazard.^35^ Violence experienced in the ED has long-term negative effects on health professionals, including loss of energy, decreased job satisfaction, anxiety, stress disorder, feelings of insecurity and depression, alcohol use disorder, smoking, suicide, and deterioration in inner life.^36^–^38^ Smoking, for example, is a significant issue among healthcare professionals, as it not only affects their physical and mental health but also undermines their role as health advocates. Bayramlar et al highlighted that medical students, as future healthcare professionals, have the potential to act as role models in tobacco control. However, their study also revealed concerning smoking prevalence rates among medical students in Istanbul, Türkiye, emphasizing the need for targeted interventions to address smoking and its associated risks in this population.^39^

Several studies have reported that among interns, females were more anxious than males.^6^,^30^,^40^,^41^ Koçak et al also found the same results in interns as we did, even though both sexes practiced in similar conditions and had the same training.^3^ This aligns with findings from Bayramlar et al, who demonstrated that female medical students exhibited higher levels of empathy and social awareness, often resulting in an increased emotional burden. These findings suggest that sex differences in anxiety may be partially explained by the greater emotional and social responsibilities usually shouldered by females in healthcare.^42^,^43^ Furthermore, studies have shown that medical interns are particularly vulnerable to depression due to the unique stressors associated with the transition into clinical practice. The adjustment to new responsibilities, fear of making medical errors, and lack of support and recognition can contribute to feelings of isolation and burnout. Additionally, stress and anxiety related to the competitiveness of medical school are closely linked to the development of depression.^27^,^33^,^44^

Another finding of interest in our study was that while females experienced higher depression and anxiety levels than males, the increases in anxiety levels was greater inr males but the increase in depression levels was the same for both sexes. Reasons for this are complicated; some studies suggest that females are more likely than their male counterparts to engage in help-seeking behaviors when facing stress and to maintain better social support networks, which facilitate active coping strategies.^45^,^46^

Interestingly, when Koçak and colleagues administered the BDI to interns at the beginning and end of their EM internship they found that depression levels had decreased by the end of the internship, which contrasts with our findings. This discrepancy may be due to differences in the internship structure, work conditions, or the specific support mechanisms available to the participants in the Koçak study. Factors such as coping mechanisms, the nature of stressors, or the overall support provided during the internship may have influenced the outcome, with some interns in the Koçak study potentially benefiting from more structured support or less exposure to high-stress situations.^3^ This highlights the complexity of mental health outcomes in EM internships and suggests that interventions aimed at reducing depression should consider these individual and situational factors.

The global COVID-19 pandemic may have increased the level of anxiety among medical interns, as the disease put both healthcare professionals and their loved ones, whom they could have infected, at risk. In this regard, Xiao et al^47^ studied healthcare workers in Wuhan, China, and other centers to determine how the COVID-19 pandemic impacted them; 55.1% of the study participants stated they experienced higher anxiety in the COVID-19 pandemic compared to the severe acute respiratory syndrome period. It was found that 54.2% showed symptoms of anxiety and 58% showed symptoms of depression.^47^ In our study, we saw no significant change in the STAI-2 scores of the participants, which indicates the trait anxiety state. There were significant decreases in the STAI-1 score, which indicates situational momentary anxiety, and in the BDI, which indicates the level of depression. This reduction may be attributed to the reduced workload in the hospital in Türkiye.^42^ Furthermore, in line with hospital policies to reduce COVID-19 exposure, shifts were temporarily halved during the pandemic period.

## LIMITATIONS

The study has several limitations that should be acknowledged. First, the single-center design and absence of a control group limit the generalizability of our findings. Additionally, the unexpected impact of the COVID-19 pandemic introduced complexity, making it difficult to isolate its effects and to distinguish pre-existing mental health symptoms from those newly developed during the pandemic. The reliance on self-reported questionnaires, such as the STAI and BDI, rather than clinical evaluations or psychiatric diagnoses, further constrains the validity of the findings.

Moreover, the study does not sufficiently explore sociodemographic factors, such as socioeconomic status, family support, and previous mental health history, which are likely to influence anxiety and depression levels. It also lacks a detailed analysis of specific stressors in EM such as violence, workload, and night shifts. It is also worth mentioning that physical activity has been associated with improvements in cognitive and academic performance, as well as reductions in depression levels. However, these findings remain a topic of discussion, and we did not explore this subject.^25^,^48^–^50^ Nor did we ask participants why they felt anxious or depressed. Understanding the reasons behind their feelings would have provided more insight into the factors contributing to their mental health challenges.

Future research should address these gaps by incorporating qualitative data, clinical evaluations, and a more detailed exploration of stress factors and sociodemographic influences to provide a more comprehensive understanding of the mental health challenges faced by healthcare workers in emergency settings.

## CONCLUSION

Our study highlights the significant psychological impact of an EM internship, showing an increase in anxiety and depression levels among medical interns, with female reporting higher levels than their male counterparts. Interestingly, depression scores did not show a significant increase in the COVID-19 group, while depression scores significantly increased in the pre-COVID-19 group by the end of their internship. Furthermore, the COVID-19 group exhibited lower post-internship STAI-1 and Beck Depression Inventory scores compared to the pre-COVID-19 group. While these results suggest an association between EM internships and increased psychological distress, it is important to note that causality cannot be definitively established due to the multifactorial nature of mental health. Although we focused on senior medical students in Turkey, the study offers valuable insights that may be applicable to medical student well-being globally. Given the increasing recognition of mental health challenges in medical education, similar studies in other countries could yield important cross-cultural comparisons and offer a broader understanding of the challenges faced by medical interns worldwide. More multicenter studies with control groups are required to better understand these relationships and guide future interventions aimed at supporting the mental well-being of medical interns.

## Supplementary Information



## Figures and Tables

**Figure f1-wjem-26-795:**
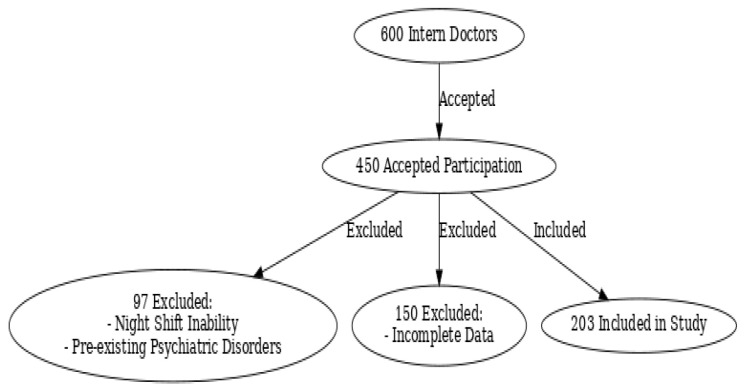
Flowchart of recruitment and selection of medical interns to participate in the study.

**Table 1:** State-Trait Anxiety Inventory and Beck Depression Inventory Scale scores by demographic information and sex.

	Male (N = 102)			Female (N = 101)			TOTAL (N = 203)			U	*P*
		
	Median	Per 25	Per 75	Median	Per 25	Per 75	Median	Per 25	Per 75		
STAI-2	40	36	47	46	40	54	44	38	51	3.495	**<.001**
BDI Pre	6	2	10	9	5	17	7	2	14	3.823	**.001**
BDI Post	9.5	4	20	14	4	25	11	4	23	4.271	**.04**
STAI-1 Pre	41	33	47	45	37	50	43	36	49	4.520	.13
STAI-1 Post	47	43	53	49	43	54	48	43	53	4.650	.23

*STAI*, State-Trait Anxiety İnventory; *BDI, Beck Depression Inventory*; *U*, Mann-Whitney U; *Per 25*, 25th percentile; *Per 75*, 75th percentile.

**Table 2:** State-Trait Anxiety Inventory and Beck Depression Inventory Scale scores before and after the internship.

		Pre-Internship (N = 203)				Post-Internship (N = 203)				Z	P
	
		Min	Mild	Mod	High	Min	Mild	Mod	High		
		
BDI	Male	74.5%	13.7%	9.8%	2.0%	50.0%	19.6%	20.6%	9.8%	14.900	**<.001**
	Female	50.5%	23.8%	22.8%	3.0%	41.6%	16.8%	27.7%	13.9%	6.092	**.01**
	Total	62.6%	18.7%	16.3%	2.5%	45.8%	18.2%	24.1%	11.8%	20.838	**<.001**
STAI_1	Male	33.3%		31.4%	35.3%	13.7%		17.6%	68.6%	21.102	**<.001**
	Female	27.7%		18.8%	53.5%	6.9%		21.8%	71.3%	12.612	**<.001**
	Total	30.5%		25.1%	44.3%	10.3%		19.7%	70.0%	33.238	**<.001**

*STAI*, State-Trait Anxiety İnventory; *BDI, Beck Depression Inventory*.

**Table 3 t3-wjem-26-795:** State-Trait Anxiety Inventory and Beck Depression Inventory Scale scores before and after COVID-19.

	Pre COVID-19 (N = 51)			COVID-19 (N = 152)			U	*P*
	
	Median	Per 25	Per 75	Median	Per 25	Per 75		
STAI-2	24	23	24	23	23	24	3.432	.22
BDI Pre	9	2	14	7	2	13	3.576	.41
BDI Post	26	15	32	8	3	16	1.348	**<.001**
STAI-1 Pre	47	38	53	41	35	48	2.999	**.02**
STAI-1 Post	51	45	56	47	42	52	2.999	**.02**

*STAI*, State-Trait Anxiety İnventory; *BDI, Beck Depression Inventory*; *Per 25*, 25th percentile; *Per 75*, 75th percentile.

**Table 4:** Risk of change in State-Trait Anxiety Inventory and Beck Depression Inventory Scale scores as a result of the internship.

		OR	Low	Upper	*P*
BDI	*Sex*	*1.145*	*0.762*	*1.721*	*.51*
	**BDI**	**1.055**	1.032	1.079	**<.001**
	*COVID-19*	*0.686*	*0.418*	*1.125*	*.14*
STAI_1	*Sex*	*1.124*	*.747*	*1.691*	*.57*
	**STAI-1**	**1.060**	1.037	1.083	**<.001**
	*COVID-19*	*0.819*	*0.510*	*1.316*	*.41*

*OR, odds ratio; STAI*, State-Trait Anxiety İnventory; *BDI, Beck Depression Inventory*.
