# Supplementary figures and images for: Effects of the COVID-19 Pandemic on Anxiety and Depression among Medical Interns
Source: West J Emerg Med. 2025 Jul 13;26(4):795–803. doi: 10.5811/westjem.38455 (PMC12342501; doi:10.5811/westjem.38455)

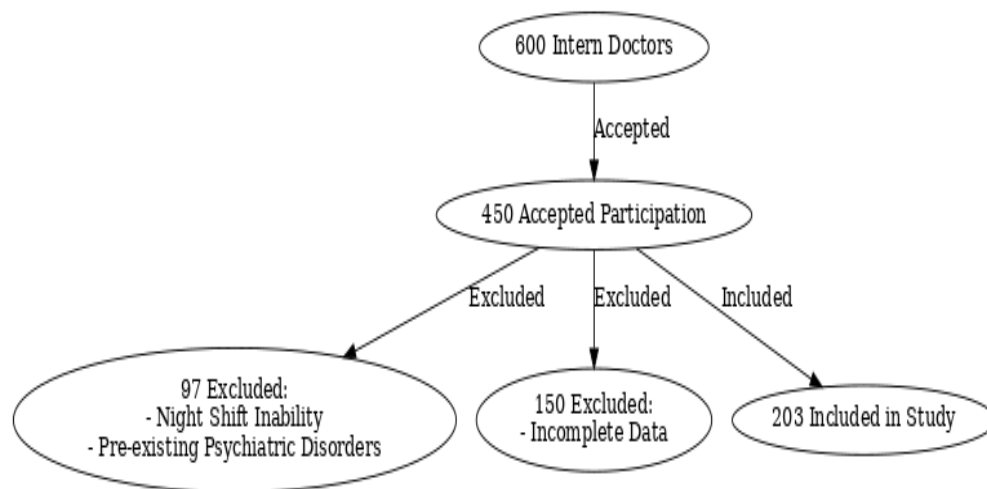

Supplement: Supplementary file 1 [file wjem-26-795-g001.pdf]
